# p38 activation induces production of miR-146a and miR-31 to repress E-selectin expression and inhibit transendothelial migration of colon cancer cells

**DOI:** 10.1038/s41598-018-20837-9

**Published:** 2018-02-05

**Authors:** Liang Zhong, Jacques Huot, Martin J. Simard

**Affiliations:** St-Patrick Research Group in Basic Oncology, CHU de Québec-Université Laval Research Centre (L’Hôtel-Dieu de Québec), Laval University Cancer Research Centre, Quebec City, Québec, G1R 3S3 Canada

## Abstract

Extravasation of circulating cancer cells determines their metastatic potential. This process is initiated by the adhesion of cancer cells to vascular endothelial cells through specific interactions between endothelial adhesion receptors such as E-selectin and their ligands on cancer cells. In the present study, we show that miR-146a and miR-181b impede the expression of E-selectin by repressing the activity of its transcription factor NF-κB, thereby impairing the metastatic potentials of colon cancer cells by decreasing their adhesion to, and migration through, the endothelium. Among the two microRNAs, only miR-146a is activated by IL-1β, through the activation of p38, ERK and JNK MAP kinases, as well as their downstream transcription factors GATA2, c-Fos and c-Jun. Inhibiting p38 MAP kinase increases NF-κB activity, at least partially via miR-146a. Inhibiting p38 also increases the expression of E-selectin at the post-transcriptional level via decreasing miR-31, which targets E-selectin mRNA and also depends on p38 for its expression. In response to IL-1β, p38 MAP kinase hence represses the expression of E-selectin at the transcriptional and the post-transcriptional levels, via miR-146a and miR-31, respectively. These results highlight novel mechanisms by which p38 downregulates the expression of E-selectin through different microRNAs following inflammatory stimuli associated to cancer progression.

## Introduction

Metastasis depends on sequential interrelated steps^1^. Notably, the adhesion of circulating cancer cells to the endothelium of blood vessels is a prerequisite for their extravasation. This adhesive event is initiated by specific interactions between endothelial adhesion receptors such as E-selectin, and their ligands on cancer cells. E-selectin is expressed exclusively by endothelial cells stimulated by pro-inflammatory cytokines including interleukin-1β (IL-1β)^2^. In an inflammatory context, E-selectin triggers the adhesion and the subsequent rolling of leukocytes on the endothelium, thus initiating their extravasation into inflamed tissues^3^. Cancer cells including breast, bladder, gastric, pancreatic and colorectal carcinoma, as well as leukemia and lymphoma can hijack this inflammatory process to extravasate and form metastases^2–4^. Accordingly, several lines of evidence suggest E-selectin as a key determinant of metastasis of colon cancer cells. In particular, the binding efficiency of colon cancer cells to E-selectin is proportional to their respective metastatic potential^5^ and an anti-E-selectin antibody is capable of reducing orthotopic liver metastasis of colon cancers^6^. The canonical model indicates that E-selectin relies on the activation of NF-κB, JNK and p38 pathways for its transcription^7–10^. However, the precise regulation of its transcription and translation following inflammatory stimuli is still largely unknown. Notably, the role of microRNAs in the signalling network governing the expression of E-selectin is ill-defined.

Among the regulators of gene expression, the evolutionarily conserved small non-coding RNA molecules called microRNAs (miRNAs) have recently emerged as key mediators of the process. To generate their functional single-stranded ~21 nucleotides long form, they are firstly transcribed as long primary miRNAs (pri-miRNAs) by RNA polymerase II. Pri-miRNAs are then processed by Drosha-DGCR8 complex in the nucleus to produce precursor miRNAs (pre-miRNAs), which are exported to the cytoplasm to be cleaved by Dicer, producing miRNAs that are loaded into miRNA-induced silencing complex (miRISC). Through base pairing with the 3′ untranslated region (3′ UTR) of mRNA, miRNA guides the miRISC to its target, thereby repressing translation with or without causing mRNA degradation^11^.

We previously reported that one of the miRNAs, miR-31, post-transcriptionally represses the expression of E-selectin by targeting its mRNA^7^. Moreover, recent reports revealed a number of miRNAs repressing the expression of E-selectin by hindering the inflammatory process. Among them, miR-146a has been shown to repress the pro-inflammatory NF-κB and JNK pathways by targeting the pro-inflammatory receptor adaptors as varied as Card10, TRAF6, IRAK1 and IRAK2, thereby deterring the expression of E-selectin^12–15^. MiR-181b also impairs the activity of the NF-κB pathway and the expression of E-selectin by targeting Card10^16^, as well as importin-α3, an importer protein required for the nuclear translocation of NF-κB^17^. MiR-10a is another miRNA impeding NF-κB-mediated E-selectin expression, through targeting two key regulators of IκBα degradation: MAP3K7 and βTRC^18^. MiR-30a represses E-selectin expression by targeting Ang2, a protein enhancing the expression of multiple adhesion receptors^19^, and miR-92a reduces E-selectin via targeting endothelial transcription factors KLF2 and KLF4^20^. However, none of these miRNAs that exhibit anti-inflammatory properties have been scrutinized in a metastatic context, to investigate their involvement in E-selectin-mediated extravasation of cancer cells.

In this study, we found that miR-146a and miR-181b inhibit NF-κB-mediated expression of E-selectin and act as potent repressors of E-selectin-dependent metastatic abilities of colon cancer cells. Among these two miRNAs, IL-1β induces only miR-146a at the transcriptional level, through p38, JNK and ERK MAP kinase pathways. Inhibiting p38 MAP kinase increases the activity of NF-κB at least partially by decreasing miR-146a. In addition, inhibiting p38 augments the expression of E-selectin at the post-transcriptional level through decreasing miR-31, a miRNA targeting E-selectin mRNA^7^.

## Results

### bmiR-146a and miR-181b repress the transcription of E-selectin

**Figure 1:**
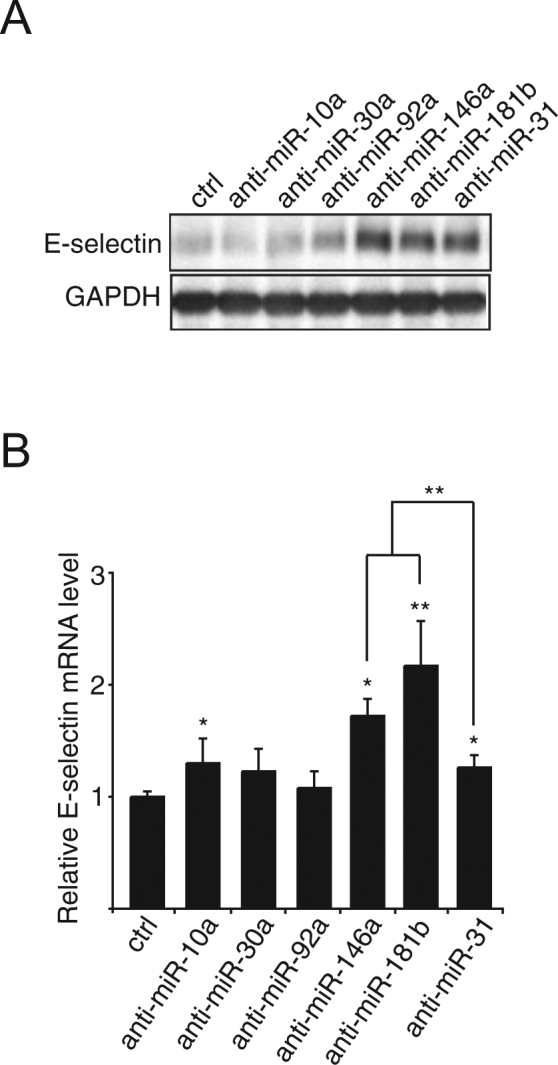
miR-146a and miR-181b repress the transcription of E-selectin. A. B. Human Umbilical Vein Endothelial Cells (HUVECs) cultivated as monolayers were transfected with 50 nM of miRNA inhibitors (anti-miR) or controls (ctrl), before being treated with IL-1β (20ng/ml) for four hours. (**A**) The expression of E-selectin was monitored by Western blotting. GAPDH was used as loading control. (**B**) RT-qPCR monitored the level of E-selectin mRNA relative to GAPDH mRNA. The Western blot is representative of four independent experiments. The quantification are the mean values of four independent experiments, the error bars represent standard errors of four independent experiments, and the significance was analyzed using a Student’s t-test. The *p*-values are calculated comparing to the ctrl, unless indicated otherwise (*p < 0.05; **p < 0.01).

To find repressors of E-selectin-dependent metastatic potentials of colon cancer cells, we first evaluated the role of miRNAs known as modulators of the inflammatory responses, namely miR-10a, miR-30a, miR-92a, miR-146a and miR-181b, in the regulation of E-selectin expression in human umbilical vein endothelial cells (HUVECs) using their respective inhibitors (henceforth anti-miRs), together with anti-miR-31 (positive control). Although anti-miR-10a mildly increased E-selectin mRNA (Fig. 1B), a corresponding increase was not observed for the protein (Fig. 1A). On the contrary, anti-miR-146a and anti-miR-181b significantly increased E-selectin expression to levels comparable to that of anti-miR-31, the inhibitor of a miRNA repressing E-selectin expression by directly targeting its mRNA^7^ (Fig. 1A). The effect of miR-146a on the regulation of E-selectin was further confirmed in human liver sinusoidal microvascular endothelial cells (HLSMECs; Supplementary Fig. S1a), but the blockage of miR-181b did not affect E-selectin level in these cells that express very high level of this miRNA (~20 fold more miR-181b detected in HLSMECs compared to HUVECs; Supplementary Fig. S1b). In HUVECs, anti-miR-146a and miR-181b also significantly increased E-selectin mRNA compared to the control and anti-miR-31 (Fig. 1B), suggesting that in contrast to miR-31, miR-146a and miR-181b may regulate E-selectin at the transcriptional level.

### miR-146a and miR-181b repress the transcription of E-selectin by inhibiting NF-κB signaling

**Figure 2:**
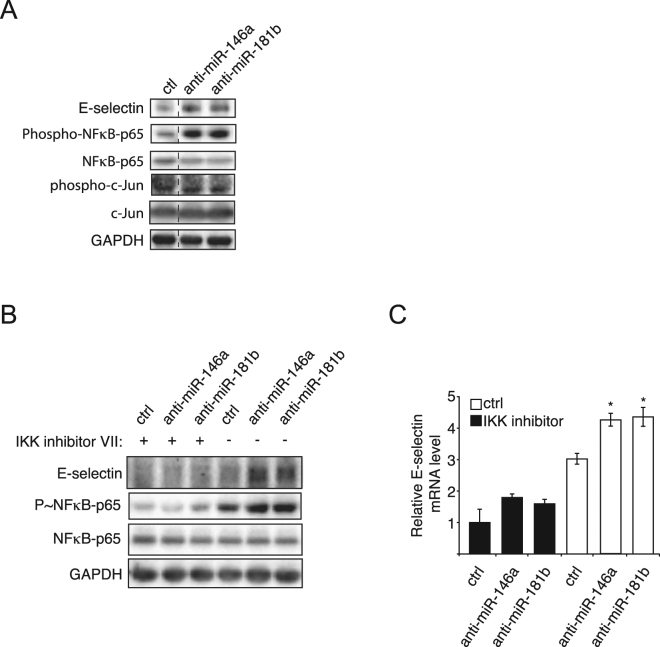
miR-146a and miR-181b repress the transcription of E-selectin by inhibiting NF-κB signalling. (**A**). Endothelial cells were treated as in Fig. 1. The activities of NF-κB and c-jun signalling pathways were monitored by Western blotting using anti-phospho-NFkB-p65 (S^536^) and anti-phospho-c-Jun (S^63^) antibodies. GAPDH was used as loading control. The dashed lines indicate that unrelated lanes have been removed between samples. See full-length blots in Supplementary Fig. S9. (**B**,**C**) Endothelial cells were transfected with 50 nM of miRNA inhibitors or control (ctrl), before being pretreated with 1 μM IKK inhibitor VII, and being treated with IL-1β (20ng/ml) for four hours. (**B**) The expression of E-selectin and the activity of NF-κB signaling pathway were monitored by Western blotting. GAPDH was used as loading control. The Western blot is representative of three independent experiments. (**C**) RT-qPCR monitored the level of E-selectin mRNA. GAPDH mRNA was used as loading control. The quantification are the mean values of three independent experiments, the error bars represent standard errors of three independent experiments, and the significance was analyzed using a Student’s t-test. The *p*-values are calculated comparing to the “anti-miR ctrl/DMSO/IL-1β” group (*p < 0.05; **p < 0.01).

MiR-146a has been reported to hamper the activation of the NF-κB and JNK pathways, two major pathways controlling the transcription of E-selectin^14^, while miR-181b particularly subdues NF-κB pathway^13,14^. To study whether the two miRNAs act on NF-κB and/or JNK pathways to affect the transcription of E-selectin, the activities of both pathways in HUVECs were evaluated using phospho-specific antibodies: anti-phospho-NF-κB-p65 (S536) and anti-phospho-c-Jun (S63)^21,22^. Both miRNA inhibitors greatly increased phosphorylated NF-κB-p65 (P~p65), but neither increased phosphorylated c-Jun (P~c-Jun) (Fig. 2A). We further investigated whether the effect of the miRNA inhibitors depends on NF-κB activity. To this end, we treated HUVECs with an inhibitor of its upstream activator IKK^23^, and found that the inhibitor greatly reduced the transcription and expression of E-selectin as well as totally abolished the ability of both miRNA inhibitors to increase them (Fig. 2B and C). These results suggest that miR-146a and −181b repress the transcription and expression of E-selectin, through inhibiting the NF-κB pathway.

### miR-146a and miR-181b modulate E-selectin-mediated adhesion to and migration through endothelial cells of colon cancer cells

**Figure 3:**
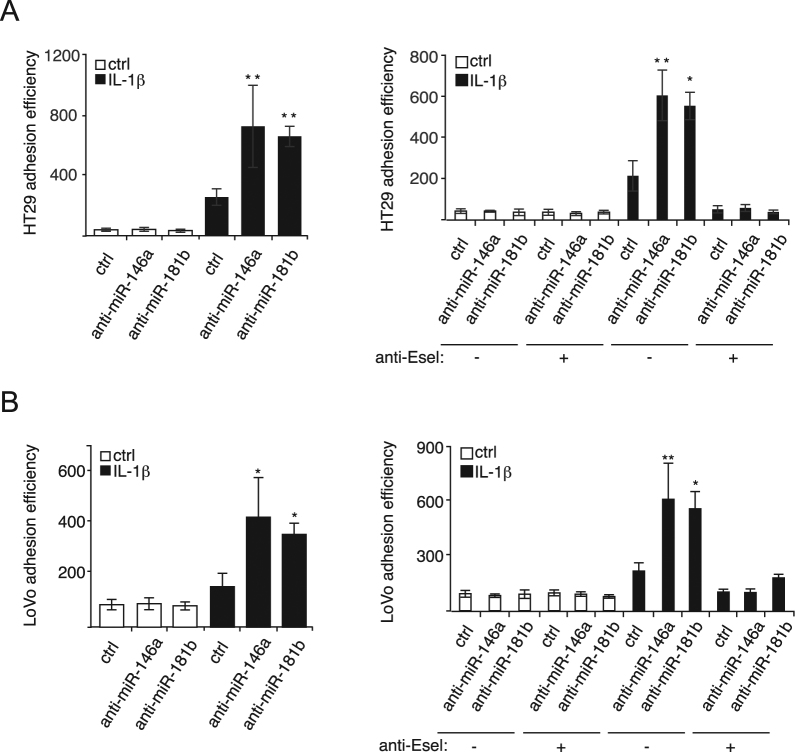
miR-146a and miR-181b modulate E-selectin-mediated adhesion of metastatic colon cancer cells to endothelial cells. (**A**) MiR-146a and miR-181b inhibit E-selectin-dependent adhesion of HT29 colon cancer cells to endothelial cells. Endothelial cells were transfected and stimulated as described in Fig. 1. Calcein AM-stained HT29 metastatic colon cancer cells were added on the tight layer of endothelial cells and incubated for 30 minutes. Non-adhering cells were washed and cells adhering to endothelial cells were determined. To test the E-selectin dependence of the effect of both miRNAs on cancer cells adhesion, anti-E-selectin antibody or MOPC21antibody (1:40) was added to endothelial cells one hour before the addition of HT29 cells. (**B**) MiR-146a and miR-181b inhibit E-selectin-dependent adhesion of LoVo colon cancer cells to endothelial cells. Experiments were carried out as described in a. Calcein AM-stained LoVo metastatic colon cancer cells were used. The quantification are the mean values of three independent experiments, the error bars represent standard errors of three independent experiments, and the significance was analyzed using a Student’s t-test. The *p*-values are calculated comparing to the “anti-miR ctrl/IL-1β(/antibody ctrl)” group (*p < 0.05; **p < 0.01). Anti-Esel.: anti-E-selectin antibody.

Adhesion of colon cancer cells to E-selectin expressing endothelial cells is a prerequisite to their transendothelial migration (TEM) during metastatic dissemination^1,24,25^. To examine whether miR-146a and miR-181b could affect the adhesion of colon cancer cells to endothelial cells, adhesion assays of two metastatic colon cancer cells, HT29 and LoVo, were performed on HUVECs transfected with miRNA inhibitors or their control. The treatment with miRNA inhibitors could not increase the adhesion of colon cancer cells when these endothelial cells were not stimulated with IL-1β and thereby did not express E-selectin^7^ (Fig. 3A and B, left panels). Moreover, treating endothelial cells with E-selectin neutralizing antibody significantly reduced the effect of miRNA inhibitors (Fig. 3A,B, right panels), indicating that the increase in the adhesion of colon cancer cells mediated by inhibiting miR-146a and miR-181b was E-selectin-dependent. Overall, these results suggest that by regulating the expression of E-selectin, miR-146a and miR-181b are important modulators of E-selectin-dependent adhesion of metastatic colon cancer cells to endothelial cells.

**Figure 4:**
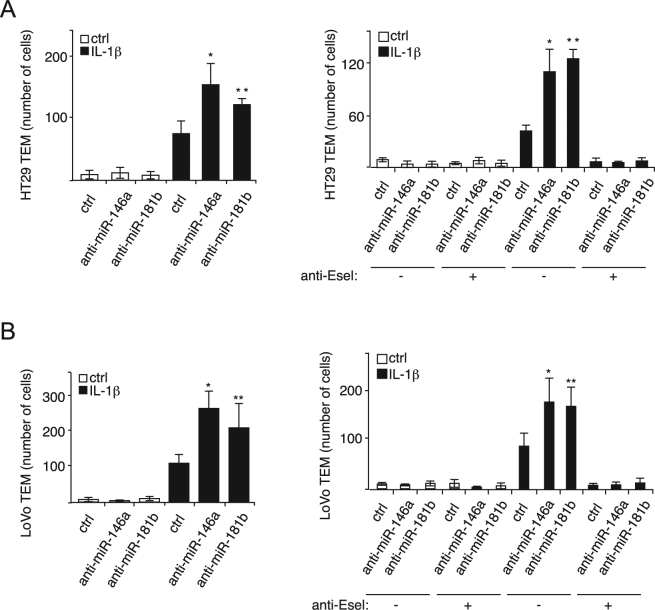
miR-146a and miR-181b modulate E-selectin-mediated migration of metastatic colon cancer cells through endothelial cells. (**A**) MiR-146a and miR-181b inhibit E-selectin-dependent TEM of HT29 colon cancer cells. In the Boyden Chambers, fluorescent HT29 cells that penetrated a tight layer of endothelial cells transfected with indicated miRNA inhibitors were counted. To test the E-selectin-dependence of the effect of both miRNAs on the TEM, anti-E-selectin antibody or MOPC21 antibody (1:40) was added to endothelial cells one hour before the addition of HT29 cells. Fluorescent cancer cells were counted with ImageJ with following parameters. Type: 8 bits, threshold: Otsu/dark background/12–255, analyze particle: 50-infinity. (**B**) MiR-146a and miR-181b inhibit E-selectin-dependent TEM of HT29 LoVo colon cancer cells. Experiments were carried out as described in a. Calcein AM-stained LoVo metastatic colon cancer cells were used. The quantification are the mean values of three independent experiments, the error bars represent standard errors of three independent experiments, and the significance was analyzed using a Student’s t-test. The *p*-values are calculated comparing to the “ anti-miR ctrl/IL-1β(/antibody ctrl)” group (*p < 0.05; **p < 0.01). Anti-Esel.: anti-E-selectin antibody.

The TEM of colon cancer cells is associated with their motile and survival potentials^26^, which are enhanced by their binding to E-selectin^27^. Since miR-146a and miR-181b are involved in modulating the expression of E-selectin and the adhesion of colon cancer cells to endothelial cells, we next studied whether they modulate the TEM of metastatic colon cancer cells by tuning their capacity to penetrate a Boyden chamber membrane coated with a tight layer of HUVECs transfected with miRNA inhibitors or their control. We first verified whether the anti-miRs could affect by themselves the integrity of the endothelial layer. The permeability to FITC dextran of endothelial cells  transfected with the anti-miRs remained similar to the endothelial layer expressing the corresponding control (Supplementary Fig. S2). This indicates that the anti-miRs do not affect by themselves the integrity of the endothelial barrier. When we inhibited miR-146a and miR-181b with anti-miRs, we observed that the TEM of both HT29 and LoVo cancer cells was increased by about two-fold (Fig. 4A,B, left panels; Supplementary Figs S3,S4) in the presence of IL-1β. Pre-treating E-selectin expressing HUVECs with anti-E-selectin antibody completely abolished the increase in TEM, supporting the essential role played by E-selectin in the process (Fig. 4A,B, right panels; Supplementary Figs S3 and S4).

Similar results of adhesion and TEM assays were obtained in HLSMECs when miR-146a was inhibited (Supplementary Figs S5 and S6). Taken all together, these results suggest that miR-146a and miR-181b are important modulators of the metastatic processes of colon cancer cells by regulating E-selectin expression.

### IL-1β induces the transcription of miR-146a via p38, JNK and ERK pathways

**Figure 5:**
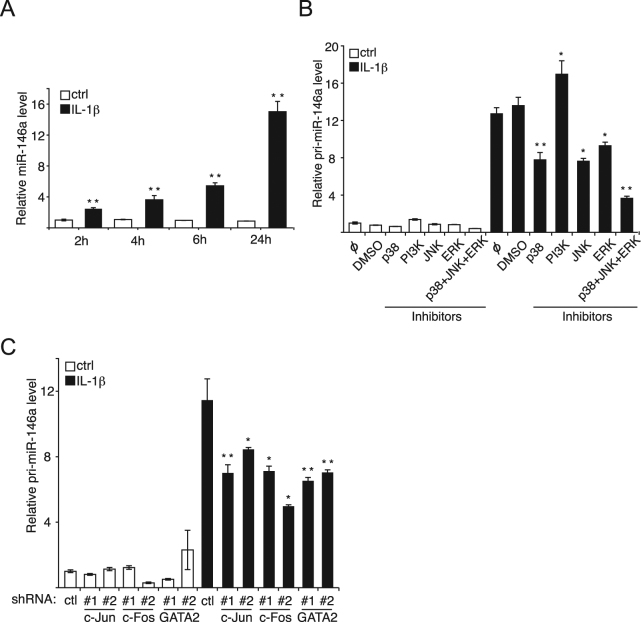
IL-1β induces the transcription of miR-146a via p38, JNK and ERK pathways. (**A**) Endothelial cells were treated with IL-1β (20ng/ml). The miR-146a level was measured by RT-qPCR and the snRNA U6 was used as the normalization control. The *p*-values are calculated comparing to the ctrl at each time point. (**B**) Endothelial cells were pre-treated with 10 μM of p38 inhibitor SB203580, 10 μM of JNK inhibitor SP600125, 10 μM of ERK inhibitor PD098059, or/and 10 μM of PI3K inhibitor LY294002 for 1 hour before the addition of IL-1β (20 ng/ml). Pri-miR-146a level was measured relative to GAPDH mRNA using RT-qPCR. The *p*-values are calculated comparing to the “DMSO/IL-1β” group. (**C**). Upon knockdown with lentivirus expressing either control (scramble shRNA) or two different shRNAs targeting each transcription factor (c-Jun, c-Fos and GATA2), pri-miR146a levels relative to GAPDH mRNA (control) were determined by RT-qPCR. The *p*-values are calculated comparing to the “shRNA ctrl/IL-1β” group. The quantification are the mean values of three independent experiments, the error bars represent standard errors of three independent experiments, and the significance was analyzed using a Student’s t-test (*p < 0.05; **p < 0.01).

Since anti-inflammatory miRNAs are often subject to regulation by pro-inflammatory cytokines, we further studied whether these miRNAs are modulated by IL-1β. Among the tested miRNAs, miR-146a was significantly induced upon exposure to IL-1β (Fig. 5A) while miR-181b was not affected in HUVECs (Supplementary Fig. S8a).

To investigate whether miR-146a is induced at the transcriptional level, pri-miR-146a was quantified with RT-quantitative PCR and we observed that its level was increased by IL-1β treatment, indicating that the transcription of miR-146a is induced (Fig. 5B). To scrutinize which pathway is responsible for the induction, a panel of kinase inhibitors were applied, and the efficiency of inhibition was validated by Western blotting (Supplementary Fig. S7a). Inhibiting p38, JNK and ERK reduced the level of pri-miR-146a by half, four hours after IL-1β treatment, and the combination of the three inhibitors further diminished it, supporting the complementary role played by the three pathways (Fig. 5B). Inhibiting PI3K mildly increased pri- and mature miR-146a (Fig. 5B; Supplementary Fig. S8b), while it significantly reduced E-selectin (Supplementary Fig. S7a). The latter observation most likely reflects the known involvement of PI3K in E-selectin transcription^9^ rather than its modest effect on miR-146a expression. These results indicate that IL-1β acts mainly through p38, JNK and ERK MAP kinases to activate the transcription of miR-146a.

We next studied which transcription factors are involved in the transcription of miR-146a. To this end, Chromatin ImmunoPrecipitation Sequencing (ChIP-seq) data of UCSC Genome Browser was used^28^. Three pro-inflammatory transcription factors were found within the regulatory regions of miR-146a gene, namely c-Jun, c-Fos and GATA2. C-Jun has been found to be activated by JNK, c-Fos is activated by ERK and p38, and GATA2 by p38^29–32^. Accordingly, lentiviral vectors expressing shRNAs were employed to efficiently silence each of these transcription factors in endothelial cells (Supplementary Fig. S7b). The knockdown of each of the three transcription factors significantly decreased the level of pri-miR-146a and thus, that of miR-146a (Fig. 5C; Supplementary Fig. S8c). Altogether, these data suggest that upon IL-1β treatment of endothelial cells, miR-146a transcription is induced through transcription factors c-Jun/c-Fos/GATA2, downstream of JNK/ERK/p38 pathways.

### p38 MAP kinase downregulates the transcription and the translation of E-selectin by modulating miR-146a and miR-31

**Figure 6:**
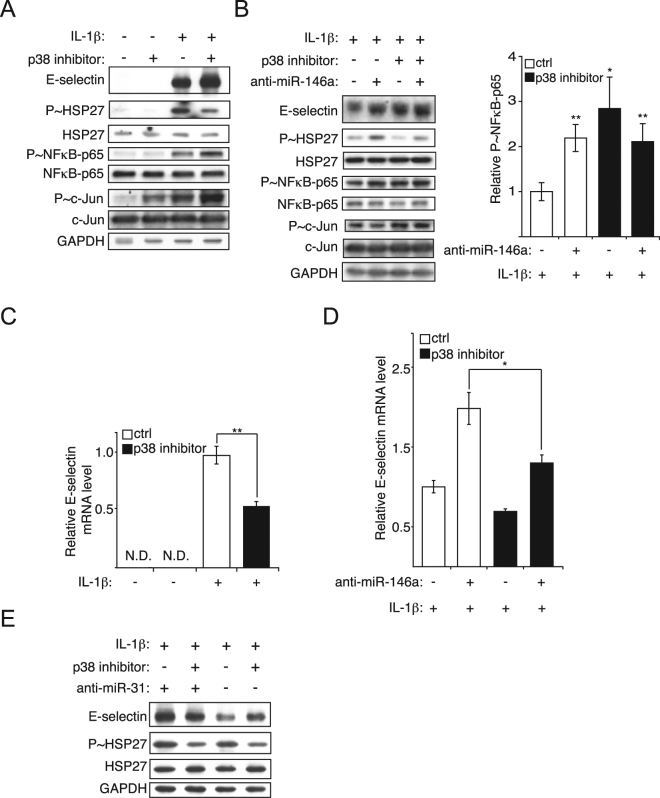
p38 MAP kinase downregulates the transcription and the translation of E-selectin by modulating miR-146a and miR-31. (**A**) Endothelial cells were pretreated with 10 μM of p38 inhibitor SB203580, before being treated with IL-1β (20ng/ml) for four hours. Western blotting monitored the expression of E-selectin and the activities of p38 (anti-phospho-HSP27 (S^82^)), NF-κB (anti-phospho-p65 (S^536^)) and c-Jun (anti-phospho-c-Jun (S^63^)) signalling pathways. GAPDH was used as loading control. (**B**) Endothelial cells were transfected with 50 nM of miRNA inhibitors or control, before being pretreated with 10 μM of p38 inhibitor SB203580, and treated with IL-1β (20 ng/ml) for four hours. Analysis as in (**A**) The *p*-values are calculated comparing to the “antagomir ctrl/DMSO/IL-1β” group. (**C**) Endothelial cells were treated as in **A**. RT-qPCR monitored the level of E-selectin mRNA relative to GAPDH mRNA. (**D**) Endothelial cells were treated as in b., then analyzed as in (**C**,**E**) Endothelial cells were treated as in (**B**) Western blotting monitored the expression of E-selectin and the activities of p38 (anti-phospho-HSP27 (S^82^) signalling pathway. GAPDH was used as loading control. The Western blots represent three independent experiments. The quantification are the mean values of three independent experiments, the error bars represent standard errors of three independent experiments, and the significance was analyzed using a Student’s t-test (*p < 0.05; **p < 0.01). N.D.: non detectable.

To reduce miRNAs hindering the expression of E-selectin and to de-repress the latter, endothelial cells were treated with different inhibitors of MAP kinases. Inhibiting p38 greatly increased the expression of E-selectin (Fig. 6A). Interestingly, this inhibition also increased P~c-Jun and P~p65 (Fig. 6A), two canonical pathways activating the transcription of E-selectin mRNA^7–10^. As miR-146a depends on p38 for its expression and is a repressor of at least NF-κB^14^, one plausible hypothesis is that p38 acts through miR-146a to repress NF-κB and the transcription of E-selectin. To test this hypothesis, endothelial cells transfected with anti-miR-146a were treated with p38 inhibitor. When miR-146a was inhibited, inhibiting p38 could no longer increase P~p65, but still increased P~c-Jun (Fig. 6B), suggesting that p38 acts through miR-146a to hinder NF-κB pathway activity, but not that of the JNK pathway. If p38 acts through miR-146a to inhibit NF-κB pathway thus impeding the expression of E-selectin, the transcription of E-selectin should be affected similarly. However, inhibiting p38 led to a decrease of E-selectin mRNA, even when miR-146a was blocked (Fig. 6C,D). This observation is in line with the report that p38 mediates the transcription of E-selectin^2–4^. Since inhibiting p38 increased E-selectin while decreasing its mRNA, p38 should repress E-selectin at the post-transcriptional level; an efficient way to promptly regulate protein level without the need of *de novo* transcription, mRNA maturation and export.

Similar to miR-146a, miR-31 also relies on p38 for its expression^7^. In addition, miR-31 inhibits the translation of E-selectin mRNA by targeting its 3′ untranslated region (3′-UTR)^7^, making a p38 - miR-31 - E-selectin post-transcriptional regulation axis conceivable. To test this model, endothelial cells were transfected with anti-miR-31 to neutralize miR-31 and rule out its regulation of E-selectin. When miR-31 was blocked, inhibiting p38 diminished E-selectin, instead of raising it (Fig. 6E), indicating that p38 acts through miR-31 to inhibit the expression of E-selectin post-transcriptionally.

## Discussion

Several lines of evidence indicate that cancer cells hijack the inflammatory system and interact with E-selectin to extravasate and form metastases. E-selectin interacts with colon cancer cells by binding to various counter-receptors constituted by a scaffold containing the Sialy Lewis a/x tetra-saccharide carbohydrate borne by signalling proteins including CD44v, CEA, PODXL, MUC16 and death receptor 3 (DR3)^2,33–37^. This interaction induces a forward signalling in endothelial cells and a reverse signalling in the cancer cells that both contribute to the TEM and extravasation of cancer cells^2,38^. The mechanisms by which E-selectin activation triggers the TEM and extravasation of cancer cells is now well documented both in *in vitro* and *in vivo* models. Notably, the group of Lubor Borsig recently showed in mouse model that the transmigration of lung cancer cells occurs in mice expressing E-selectin via monocyte-mediated endothelial activation, which is associated with lung metastasis^39^. In contrast, no metastasis occurs in E-selectin −/− mice. Moreover, depletion of monocytes prevents an increase in lung vascular permeability in E-selectin expressing mice. They proposed that E-selectin-dependent TEM of cancer cells results from dissociation of VE-cadherin junctions and actin retraction, both as a result of E-selectin activation by CCL2 released by monocyte binding to E-selectin^39^. Interestingly, this confirms the results we obtained in earlier studies in which we show that the endothelial activation of E-selectin by clustering antibodies triggers TEM via dissociation of VE-cadherin/β catenin complex at adherens junctions in addition to p38-mediated actin retraction^40^.

Despite the importance of E-selectin in metastatic progression, little is known about the mechanisms that downregulate its expression and stop the E-selectin-mediated adhesion process. Here, we show that miR-146a and miR-181b repress the expression of E-selectin by inhibiting the NF-κB pathway that controls the transcription of E-selectin. By doing so, miR-146a and miR-181b-mediated repression of E-selectin impairs the metastatic potentials of colon cancer cells by decreasing their adhesion to, and migration through, the endothelium.

**Figure 7:**
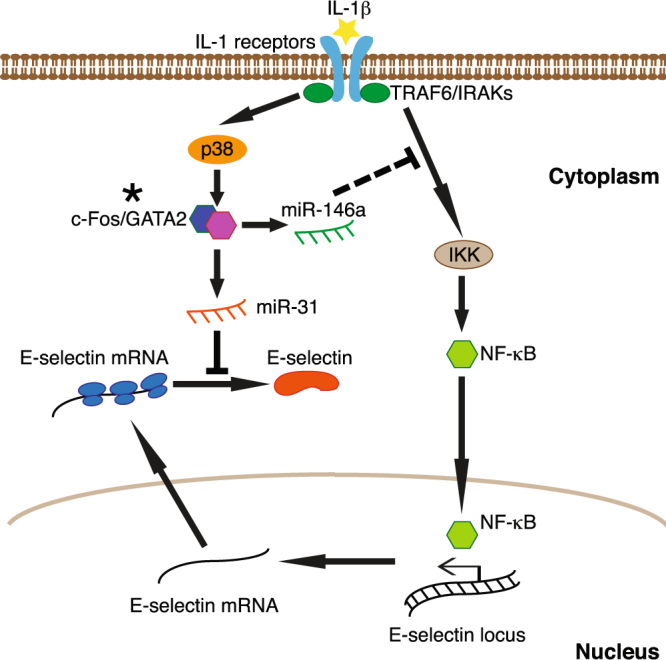
p38 mediates the transcription and the translation of E-selectin by modulating miR-146a and miR-31. Following stimulation with IL-1β, the receptor-adaptor complex signals through NF-κB pathway to activate the transcription of E-selectin. In parallel, the complex also signals through p38 pathway to induce the production of miR-146a and miR-31. The former, in a feedback loop, targets adaptor proteins TRAF6 and IRAKs to repress NF-κB-dependent E-selectin expression at the transcriptional level. The latter targets E-selectin mRNA to inhibit its translation. *In order to simplify the figure, the transcription of miRNAs is presented in the cytoplasm.

MiR-146a is induced by IL-1β at the transcriptional level in a process involving p38, ERK and JNK MAP kinases and their downstream transcription factors c-Jun, c-Fos and GATA2. In addition, inhibiting p38 leads to decreased miR-146a, thus de-repressing the NF-κB pathway. However, due to the implication of transcription factors other than NF-κB in transcribing E-selectin downstream of p38, this de-repression is not transmitted into corresponding mRNA level change^10^. Inhibiting p38 de-represses the expression of E-selectin at the post-transcriptional level via miR-31, which also depends on p38 for its expression, and inhibits the translation of E-selectin mRNA^7^ (Fig. 7).

MiR-146a has been reported to target the pro-inflammatory receptor adaptors Card10, TRAF6, IRAK1 and IRAK2. It thus has the potential to repress the pro-inflammatory NF-κB, ERK and JNK pathways^14^, among which NF-κB and JNK mediate the transcription of E-selectin^9,10^. According to our results, miR-146a has detectable effect only on NF-κB pathway, but not on JNK pathway, suggesting the involvement of rather the former in miR-146a-mediated repression of E-selectin expression but not the latter. In addition, our observations are in line with the claim that miR-181b inhibits the activity of the NF-κB pathway by targeting Card10 and importin-α3^16^.

The conclusion that miR-146a regulates the expression of E-selectin is supported by three independent but complementary observations. First, IL-1β-induced expression of miR-146a is inversely correlated with that of E-selectin, being maximal at 24 h, whereas E-selectin decreases to almost null level 24 hours after stimulation^7^. Second, inhibiting miR-146a is associated with an increase of E-selectin. Last, inhibiting miR-146a enhances the activity of NF-κB, upon which E-selectin mRNA is transcribed. Accordingly, inhibition of NF-κB totally abolished the ability of anti-miR-146a to increase E-selectin. MiR-181a, although cannot be induced by IL-1β, also exhibits the ability to hamper NF-κB-mediated E-selectin expression at least in HUVECs. On the contrary, our results indicate that inhibiting miR-10a, miR−30a or miR-92a is not associated with E-selectin expression.

One novelty of our study is the identification of p38, ERK and JNK as major pathways regulating the expression of miR-146a. This is supported by the observation that the three pathways are induced by IL-1β and that their inhibition impairs the IL-1β-induced miR-146a. The regulation occurs at the transcriptional level given that: (1) IL-1β increased the level of pri-miR-146a; and (2) a decreased level of miR-146a in the presence of inhibitors is accompanied by a reduction of pri-miR-146a. ChIP-seq data from UCSC Genome Browser indicate that c-Jun, c-Fos and GATA2 are pro-inflammatory transcription factors bound to regulatory sites of the genome of miR-146a. They can act as transcription factors regulating miR-146a following IL-1β stimulation. Along these lines, the knockdown of c-Fos, c-Jun and GATA2 in endothelial cells reduced the IL-1β-induced transcription and production of miR-146a. Intriguingly, these transcription factors are all known to be activated by the three kinases, of which c-Jun can be activated by JNK, c-Fos can be activated by ERK and p38, and GATA2 can be activated by p38. It has been reported that AP1 (heterodimer of c-Jun and c-Fos) and GATA2 can act cooperatively to regulate transcription, which is in alignment with our data^41^.

Another important observation is that inhibiting p38 can de-repress NF-κB pathway by down-regulating miR-146a. The phosphorylation of c-Jun is also augmented by this inhibition, but as miR-146a does not affect phosphorylated c-Jun in our experiments, the mechanism still remains unknown. One plausible explanation is that, following stimulation by IL-1β, when one pathway such as p38 is inhibited, the activity of other pathways might be increased to compensate for the lost signal transduction. In any scenario, miR-146a plausibly acts as a bridge connecting NF-κB and p38 pathways to ensure signal transduction. Inhibiting p38 pathway also increased E-selectin without augmenting its mRNA, suggesting a post-transcriptional regulation. Further research revealed miR-31, another miRNA regulated by the p38 pathway that targets E-selectin mRNA^7^, as responsible for this de-repression of E-selectin. Since p38-ATF2 axis is one of the three major pathways controlling the transcription of E-selectin mRNA^10^, increased c-Jun activity by a yet unknown mechanism and de-repressed NF-κB activity by reduced miR-146a do not play a major role in the de-repressed E-selectin expression upon inhibiting p38 MAP kinase. Hence, in addition to modulate the transcription of E-selectin directly, by mediating also the transcription of two miRNAs repressing E-selectin at two different levels (i.e. transcription and translation), p38 tightly controls the expression of this important cell adhesion molecule (Fig. 7). Previous studies have shown that after 4-6 hours the expression of E-selectin at the cell surface decreases following its internalization and degradation in the lysosomes^8^. Our findings now highlight new mechanisms by which the expression of E-selectin is precisely down-regulated by miRNAs.

In accordance with our finding that miR-146a and miR-181b regulate the expression of E-selectin, inhibiting them is associated with increased adhesion and migration of metastatic colon cancer cells to, and through, endothelial monolayer, both of which are abolished by E-selectin neutralizing antibody. These findings support our argument that miR-146a and miR-181b have anti-metastatic properties against colon cancers. In the long run, miR-146a and miR-181b may be proved as key players in the metastatic process of not only colorectal carcinoma, but also breast, bladder, gastric, and pancreatic carcinoma, leukemia and lymphoma, which all depend on E-selectin for their extravasation^2–4^. Our data are the first to report the miRNA modulation of E-selectin expression and functions in an *in vitro* model of TEM of cancer cells. Accordingly, we believe that our results are important and hope they will pave the road to *in vivo* studies. On the other hand, evidence is available for miRNA regulation of E-selectin during inflammatory diseases such as atherosclerosis^38^. Given that E-selectin is expressed in response to inflammation, we believe that the results in support of miRNA/E-selectin as mediators of the inflammatory process *in vivo* should soon be translated into *in vivo* models of diseases associated with inflammation including the tumor initiation and progression.

In conclusion, our study supports the critical role played by miRNAs in regulating E-selectin dynamics. Notably, it highlights the role of miR-146a and miR-181b as transcriptional modulators regulating E-selectin and E-selectin-dependent metastatic abilities of colon cancer. Furthermore, our findings raise the possibility that both miRNAs may be maintained at a low level in endothelial cells constantly expressing E-selectin, thus promoting the transendothelial migration of cancer cells during metastasis. In this context, increasing their expression in endothelial cells may be envisioned as an approach to reduce metastases of cancer cells which extravasate in an E-selectin-dependent manner into organs as various as liver, bone marrow, skin and lung^2,4^. In corollary, it might be expected that a low level of miR-146a and/or miR-181b in endothelial cells could serve as a biomarker of pro-metastatic states.

## Methods

### Reagents

Chemical inhibitors PD098059, LY294002, SP600125 and SB203580 were obtained from Sigma (St Louis, MO). IKK Inhibitor VII was obtained from EMD Millipore (Billerica, MA). CHIR99021 was obtained from Thermo Fisher (Montreal, QC). Calcein-AM was obtained from Invitrogen Molecular Probes (Burlington, ON). Dimethylsulfoxyde was purchased from Thermo Fisher Scientific (Montreal, QC). IL-1β was obtained from R&D Systems (Minneapolis, MN).

### Cells

Human umbilical vein endothelial cells (HUVECs) were isolated by collagenase digestion of umbilical veins from undamaged fresh cords, as described^42^. This human material was obtained in accordance with the relevant guidelines and regulations. In that regard, the authorization of the human ethical committee of the CRCHU de Québec-Université Laval was obtained to do the study and participating mothers signed informed consents. HUVECs at passages ≤ 4 were grown to form monolayer in EGM2 endothelial cell growth medium (Lonza, Allendale, NJ) in gelatin-coated tissue culture flasks. Human liver sinusoidal microvascular endothelial cells (HLSMECs) and its Complete Classic Medium With Serum and CultureBoost were purchased from Cell Systems (Kirkland, WA) and grown using the same protocol as for HUVECs. HT29 (ATCC) colorectal adenocarcinoma cells were cultivated in McCoy 5 A medium (Sigma, St Louis, MO) supplemented with 10% [v/v] foetal bovine serum (FBS). LoVo (ATCC) colorectal adenocarcinoma cells were cultivated in Ham’s F12 nutrient mixture (Thermo Fisher Scientific, Carlsbad, CA) supplemented with 10% [v/v] FBS. All cells were cultivated at 37 °C in 5% CO_2_ humidified atmosphere.

### shRNAs and miRNA inhibitors

MiRIDIAN miRNA hairpin inhibitors and negative control #1 were obtained from Dharmacon (Lafayette, CO). These miRNA inhibitors bind to miRNAs by complementary sequences and thus block their capacity to silence mRNA target without affecting the level of miRNAs^43^. Lentiviral particles containing shRNAs against c-Jun (TRCN0000039589, TRCN0000039590), c-Fos (TRCN0000016004, TRCN000016007) and GATA2 (TRCN0000019264, TRCN0000019265) were obtained from Sigma (St Louis, MO).

### Transfection and infection

Endothelial cells were transfected using X-tremeGene HP Transfection Reagent following manufacturers’ protocol (Roche Life Science, Laval, QC). Endothelial cells were infected by lentivirus in the presence of 8 μg/mL hexadimethrine bromide (Sigma, St Louis, MO).

### RNA extraction and quantification

Cells were lysed in TRIzol (Thermo Fisher Scientific, Carlsbad, CA) to extract total RNA following manufacturer’s protocol. The quality of RNA was assessed on agarose gel and spectrometry. The reverse transcription was performed with TaqMan miRNA Reverse Transcription Kit (Thermo Fisher Scientific, Carlsbad, CA) using specific primers (Thermo Fisher Scientific, Carlsbad, CA) for miRNAs, or random primers for pri-miRNAs. Sybr Green was carried out with iScript reverse transcription supermix and SsoAdvanced universal Sybr Green supermix from Bio-Rad (Hercules, CA). The qPCR was carried out with Universal PCR Master Mix, (Thermo Fisher Scientific, Carlsbad, CA) and specific probes from the same company. For miR-181b, its reverse transcription was performed with TaqMan Advanced miRNA cDNA Synthesis Kit (Thermo Fisher Scientific, Carlsbad, CA), and its qPCR was carried out with TaqMan Fast Advanced Master Mix from the same company (Thermo Fisher Scientific, Carlsbad, CA)following manufacturer’s protocol.

### Western blotting

Cells were lysed using SDS-PAGE loading buffer without reducing agents. Proteins were separated by SDS-PAGE and transferred to a nitrocellulose membrane. Antibodies were applied according to their manufacturers’ protocols. Blots were developed with SuperSignal West Pico Substrate (Thermo Fisher Scientific, Montreal, QC). Anti-phospho-ERK1/2 MAPK (T^202^/Y^204^) mouse antibody (1:1000), anti-ERK1/2 MAPK mouse antibody (1:1000), anti-phospho-Akt (S^473^) rabbit antibody (1:1000), anti-Akt rabbit antibody (1:1000), anti-phospho-c-Jun (S^63^) rabbit antibody (1:1000), anti-c-Jun rabbit antibody (1:1000), anti-phospho-p65 (S^536^) rabbit antibody (1:1000), anti-p65 rabbit antibody (1:1000), anti-phospho-HSP27 (S^82^) rabbit antibody (1:1000) and anti-HSP27 rabbit antibody (1:1000) were obtained from Cell Signaling Technology (Beverly, MA), Anti-E-selectin mouse monoclonal antibody (1:1000) was obtained from R&D Systems (Minneapolis, MN). Anti-METTL3 rabbit antibody (1:2000) was obtained from Abcam (Cambridge, UK). Anti-GAPDH mouse antibody (1:10000) was obtained from Novus Biologicals (Oakville, ON). Anti-mouse/rabbit-IgG-horseradish-peroxidase (HRP) goat antibodies (1:5000) were obtained from The Jackson Laboratory (Bar Harbor, ME).

### Adhesion assay

Endothelial cells were plated on gelatin-coated wells and left to grow to confluence. HT29 and LoVo cells were labeled with calcein-AM for 30 min at 37 °C, then were added to endothelial cells for 30 min. The endothelial layer was washed twice with PBS and the attached cells were quantified by measuring the fluorescence emission with Fluoroskan Ascent™ Microplate Fluorometer (Thermo Fisher Scientific, Carlsbad, CA). To study the E-selectin-dependence of the adhesion, neutralizing anti-E-selectin mouse antibody (1:5000, Cedarlane Labs (Burlington, ON)) or the control MOPC21 antibody (1:40, Abcam (Cambridge, UK)) was introduced one hour before adding HT29 or LoVo cells.

### Endothelial permeability assay

The endothelial barrier integrity was evaluated by measuring the permeability across endothelial cell monolayers of FITC-Dextran in a gelatin-coated Transwell units (Boyden chambers 6.5 mm diameter, 0.4 μm pore size polycarbonatefilter]; Corning, Pittston, PA]. More precisely, endothelial permeability was determined by measuring the passage of FITC-labelled dextran (1 mg/ml of fluorescein isothiocyanate-dextran, molecular mass: 40 kDa; Sigma, St Louis, MO) through the endothelial monolayer during 30 minutes. 100 μl was collected from the lower compartment and fluorescence was evaluated using a Fluoroskan Ascent Microplate Fluorometer following manufacturer’s protocol (Thermo Fisher Scientific, Montreal, QC).

### Transendothelial migration assay

Cell migration was investigated using a modified Boyden Chamber assay. Endothelial cells were grown to confluence on a 5.0 μm-pore-sized gelatinized polycarbonate membrane separating the two compartments of a 6.5 mm migration chamber (Corning, Pittston, PA). After IL-1β-mediated activation of endothelial cells for 4 hours, calcein-AM stained HT29 or LoVo cells suspended in migration buffer (medium199, 10 mM HEPES pH7.4, 1.0 mM MgCl_2_, 0.5% [w/v] BSA) were added to the monolayer of endothelial cells, previously washed with the same buffer. After five hours, cells on the upper face of the membrane were scraped with a cotton swab. The number of HT29 or LoVo cells that have migrated to the lower face of the filter was counted using an inverted fluorescence microscope. To study the E-selectin-dependence of the migration, neutralizing anti-E-selectin mouse antibody (1:5000, Cedarlane Labs (Burlington, ON)) or the control MOPC21 antibody (1:40, Abcam (Cambridge, UK)) was introduced one hour before adding HT29 or LoVo cells.

## Electronic supplementary material

Supplementary Figures
