# Supplementary material for: p38 activation induces production of miR-146a and miR-31 to repress E-selectin expression and inhibit transendothelial migration of colon cancer cells
Source: Sci Rep. 2018 Feb 5;8:2334. doi: 10.1038/s41598-018-20837-9 (PMC5799178; doi:10.1038/s41598-018-20837-9)
Supplement: Supplementary file 1 — Supplementary Figures [file 41598_2018_20837_MOESM1_ESM.pdf]

p38 activation induces production of miR-146a and miR-31 to repress E-selectin expression and inhibit transendothelial migration of colon cancer cells

Liang Zhong, Jacques Huot*, Martin J. Simard*

Supplementary Figures

Figure S1.

miR-146a represses the transcription of E-selectin in HLSMECs. A. HLSMECs cultivated as monolayers were transfected with 50nM of miRNA inhibitors or controls, before being treated with IL-1β (20ng/ml) for four hours. The expression of E-selectin was monitored by Western blotting. GAPDH was used as loading control. **B.** HLSMECs and HUVECs were treated with IL-1β (20ng/ml) for four hours. The miR-146a level was measured by RT-qPCR and the snRNA U6 was used as the normalization control. The Western blot is representative of four independent experiments. The quantifications are the mean values of three independent experiments, the error bars represent standard errors of three independent experiments, and the significance was analyzed using a Student's t-test. The *p*-values are calculated comparing to the ctrl, unless indicated otherwise (*p <0.05; **p<0.01).

Figure S2.

Inhibiting miR-146a and miR-181b does not change the integrity of HUVEC monolayer. A. Images of endothelial cells transfected with miRNA inhibitors or control. **B.** The permeability of HUVECs transfected with miRNA inhibitors or control to FITC dextran. FITC dextran (1 mg/ml) was added to Boyden Chambers coated with indicated endothelial cells for 30 minutes. The quantifications are the mean values of three independent experiments, the error bars represent standard errors of three independent experiments, and the significance was analyzed using a Student's t-test.

Figure S3.

miR-146a and miR-181b modulate E-selectin-mediated adhesion to and migration through HUVECs of HT29 colon cancer cells. Images of transmigrated fluorescent HT29 colon cancer cells. Images are representative of three independent experiments. Anti-Esel.: anti-E-selectin antibody.

Figure S4.

miR-146a and miR-181b modulate E-selectin-mediated adhesion to and migration through HUVECs of LoVo colon cancer cells. Images of transmigrated fluorescent LoVo colon cancer cells. Images are representative of three independent experiments. Anti-Esel.: anti-E-selectin antibody.

Figure S5.

miR-146a modulates E-selectin-mediated adhesion and transendothelial migration of metastatic colon cancer cells to and through HLSMECs. A. Mir-146a inhibits E-selectin-dependent adhesion of HT29 colon cancer cells to HLSMECs. The experiments were carried out as in Figure 3 except that HLSMECs were used instead of HUVECs. **B.** Mir-146a inhibits E-selectin-dependent transendothelial migration (TEM) of HT29 colon cancer cells through HLSMECs. The experiments were carried out as in Figure 4 except that HLSMECs were used instead of HUVECs. The quantifications are the mean values of three independent experiments, the error bars represent standard errors of three independent experiments, and the significance was analyzed using a Student's t-test (*p <0.05; **p<0.01). Anti-Esel.: anti-E-selectin antibody.

Figure S6.

miR-146a and miR-181b modulate E-selectin-mediated transendothelial migration of metastatic colon cancer cells through HLSMECs. Images of transmigrated fluorescent HT29 colon cancer cells. Images are representative of three independent experiments. Anti-Esel.: anti-E-selectin antibody.

Figure S7.

IL-1β induces the production of miR-146a via p38, JNK and ERK pathways. A. The inhibition of p38, JNK, PI3K and ERK pathways were confirmed by Western blotting with antiphospho-HSP27 (S82), anti-phospho-c-Jun (S63), anti-phospho-Akt (S473) and anti-phospho-ERK1/2 (T202/Y204) antibodies, respectively. **B.** The knockdowns of each transcription factor were validated by Western blotting. The Western blots represent three independent experiments.

Figure S8.

IL-1β induces the transcription of miR-146a via p38, JNK and ERK pathways. miR-181b and -146a levels were measured by RT-qPCR and the snRNA U6 was used as the normalization control. The quantification are the mean values of three independent experiments, the error bars represent standard errors of three independent experiments, and the significance was analyzed using a Student's t-test (*p <0.05; **p<0.01).

Figure S9.

Full-length blots for Fig. 2A.

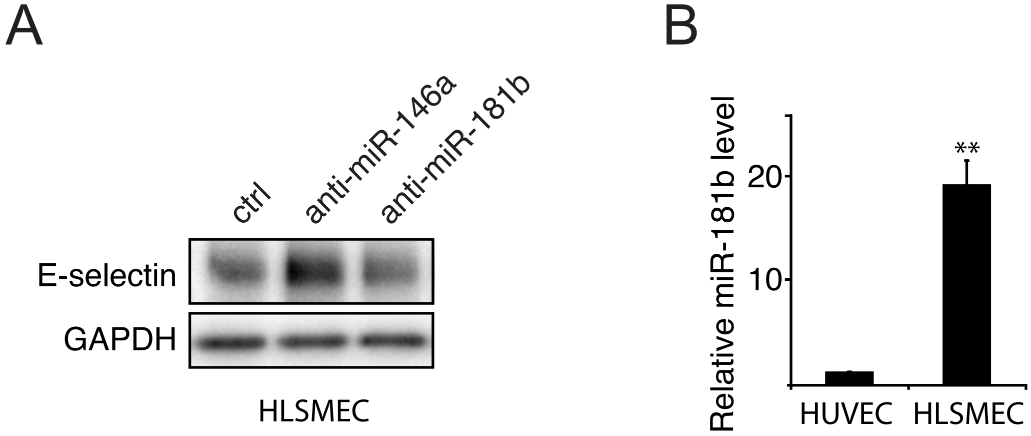

Zhong et al Figure S1

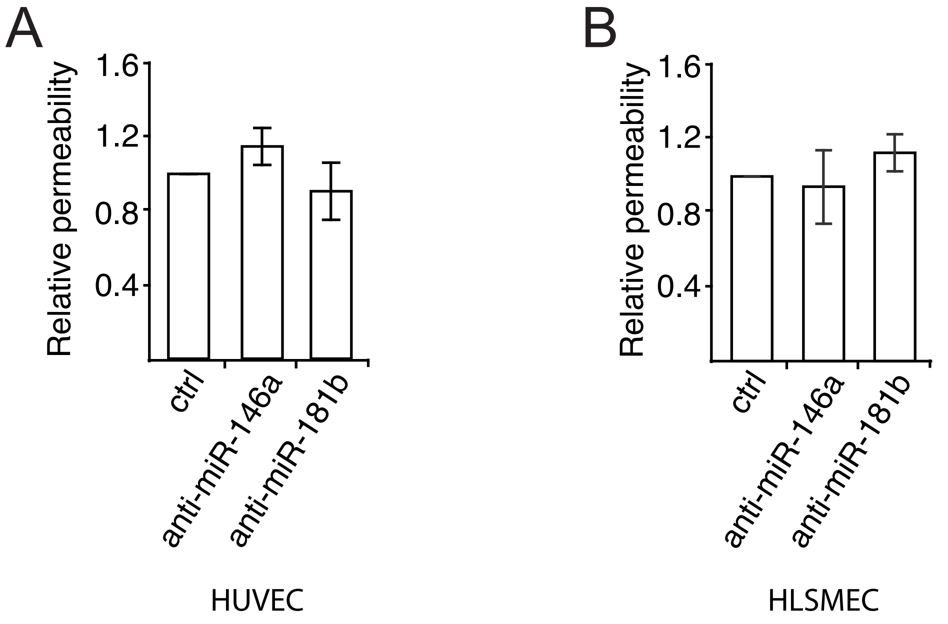

Zhong et al Figure S2

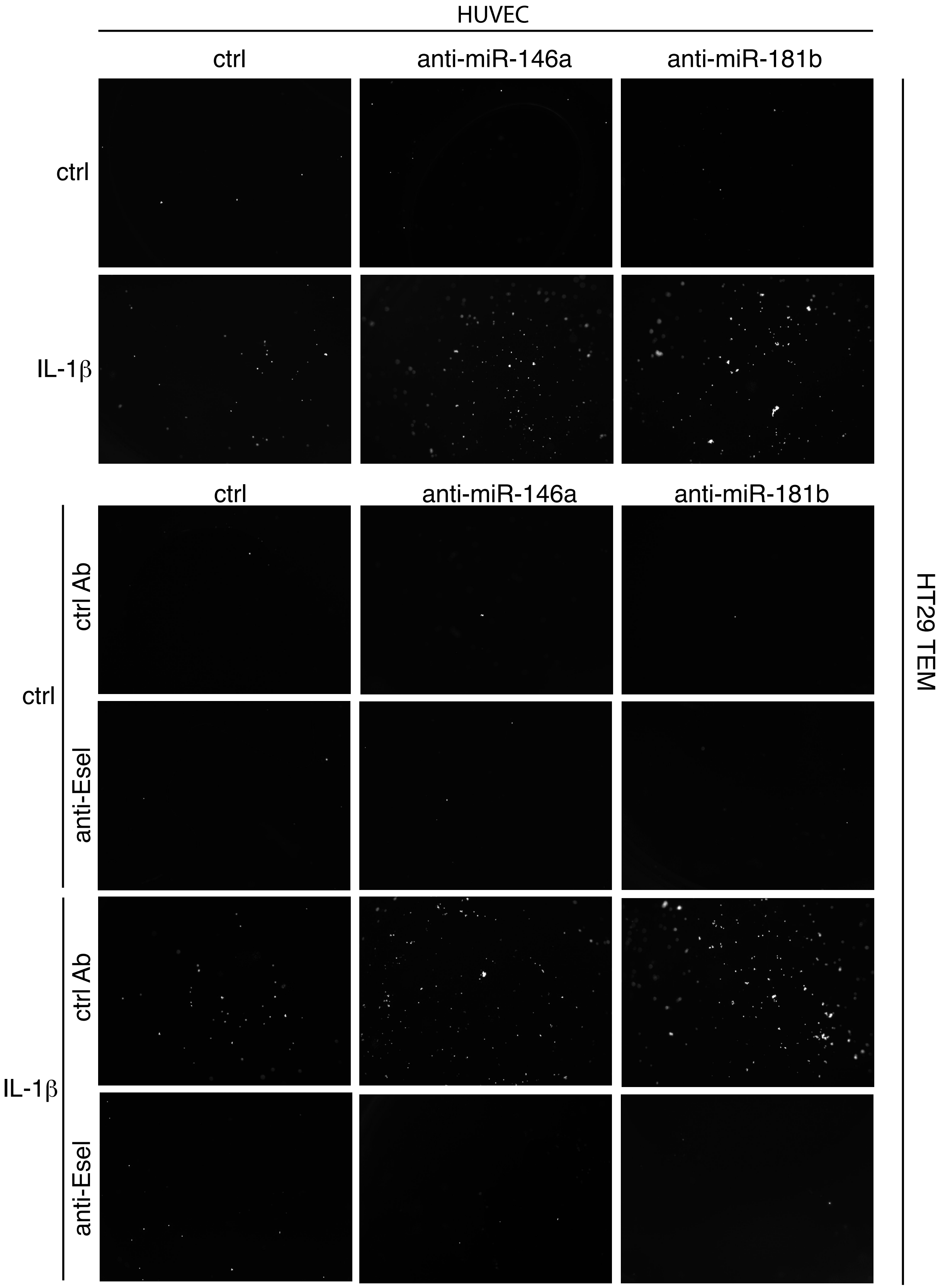

Zhong et al Figure S3

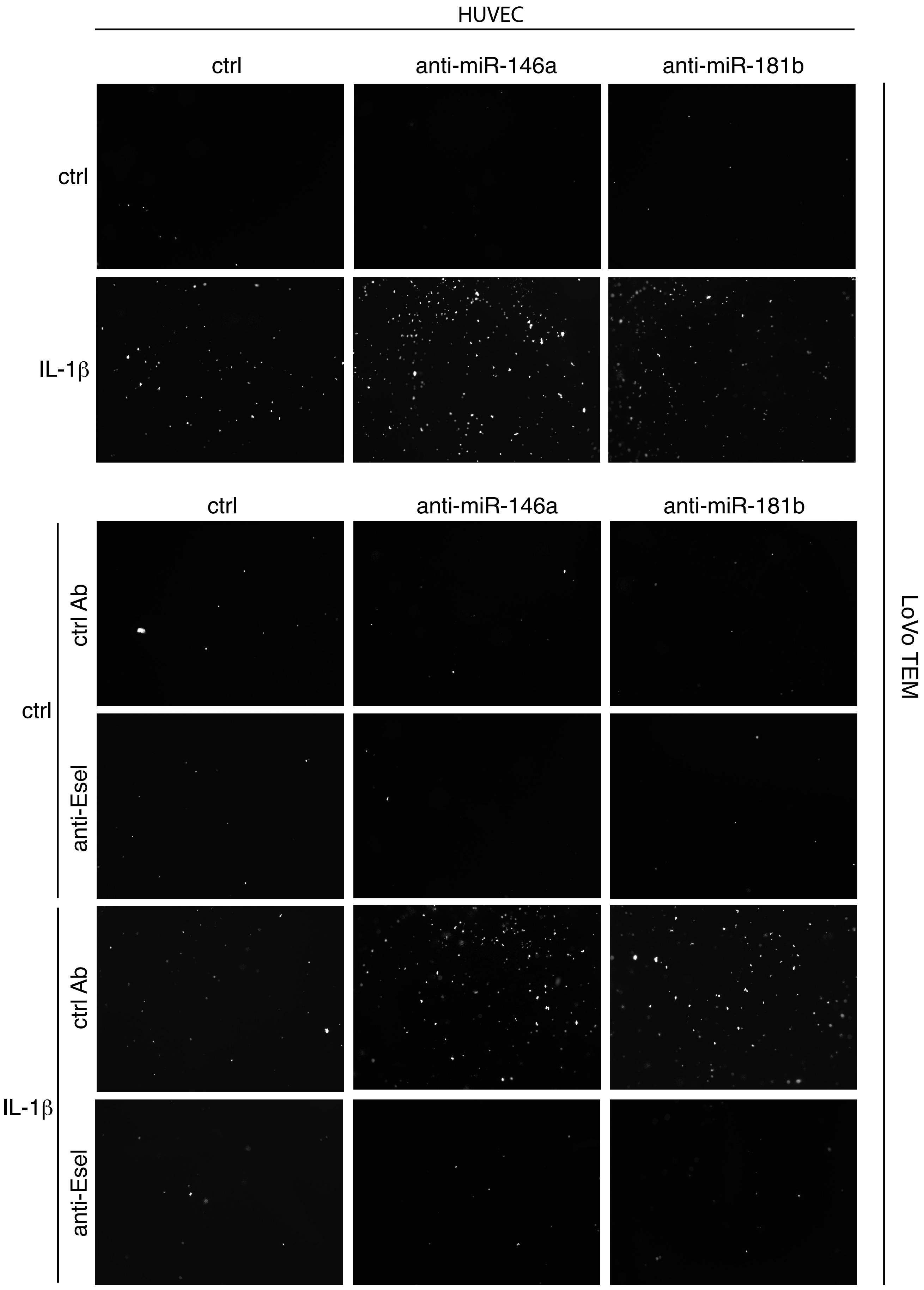

Zhong et al Figure S4

A

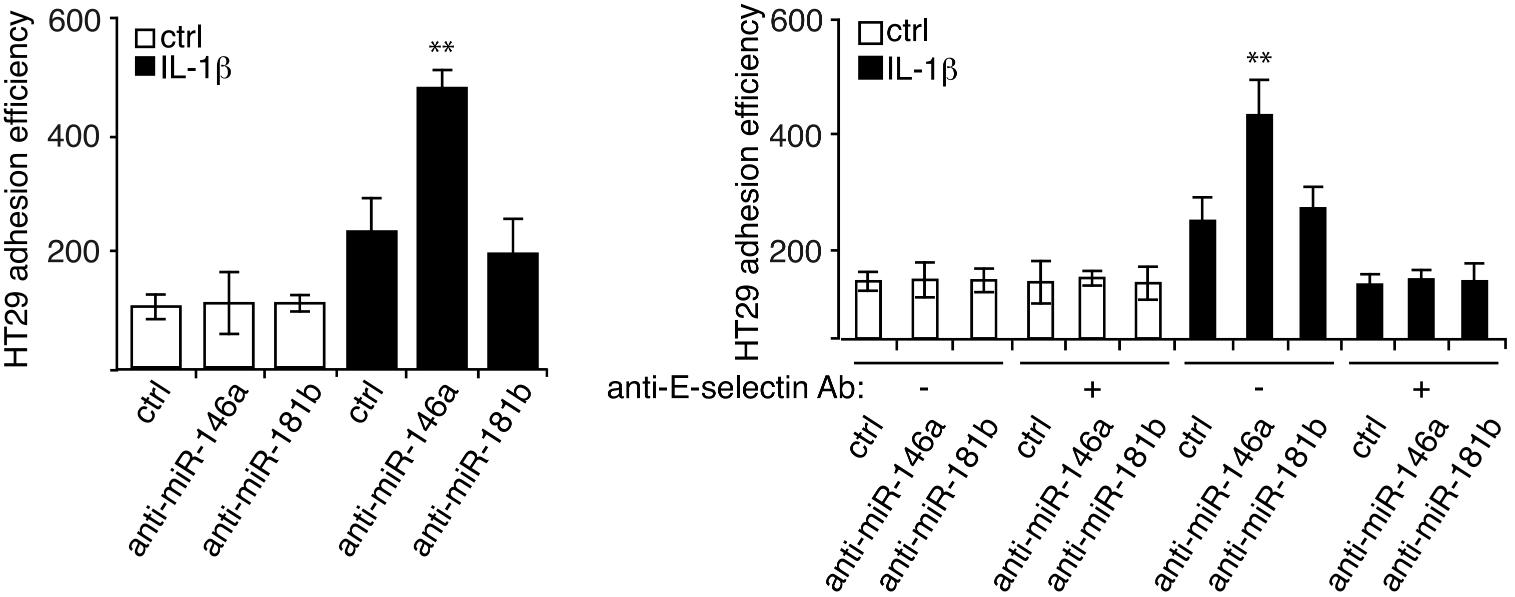

B

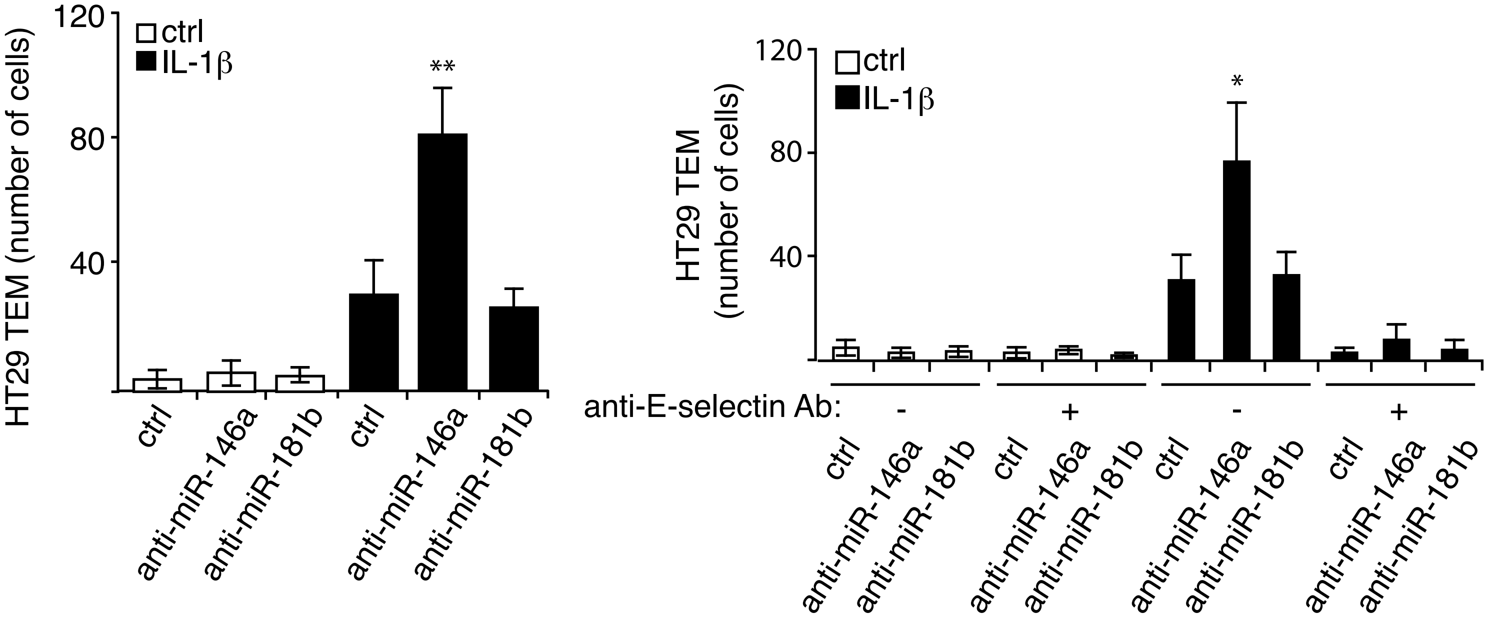

Zhong et al Figure S5

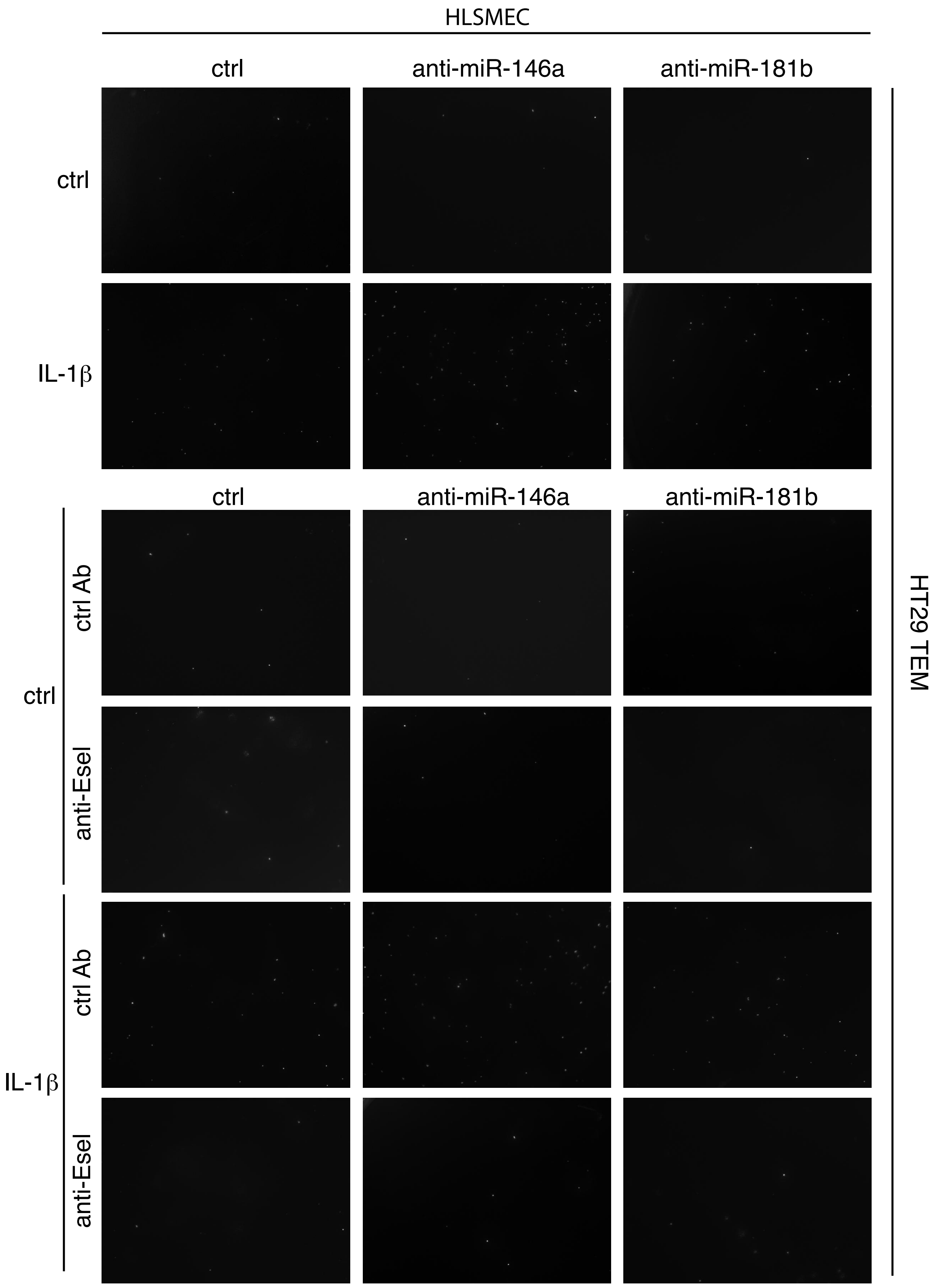

Zhong et al Figure S6

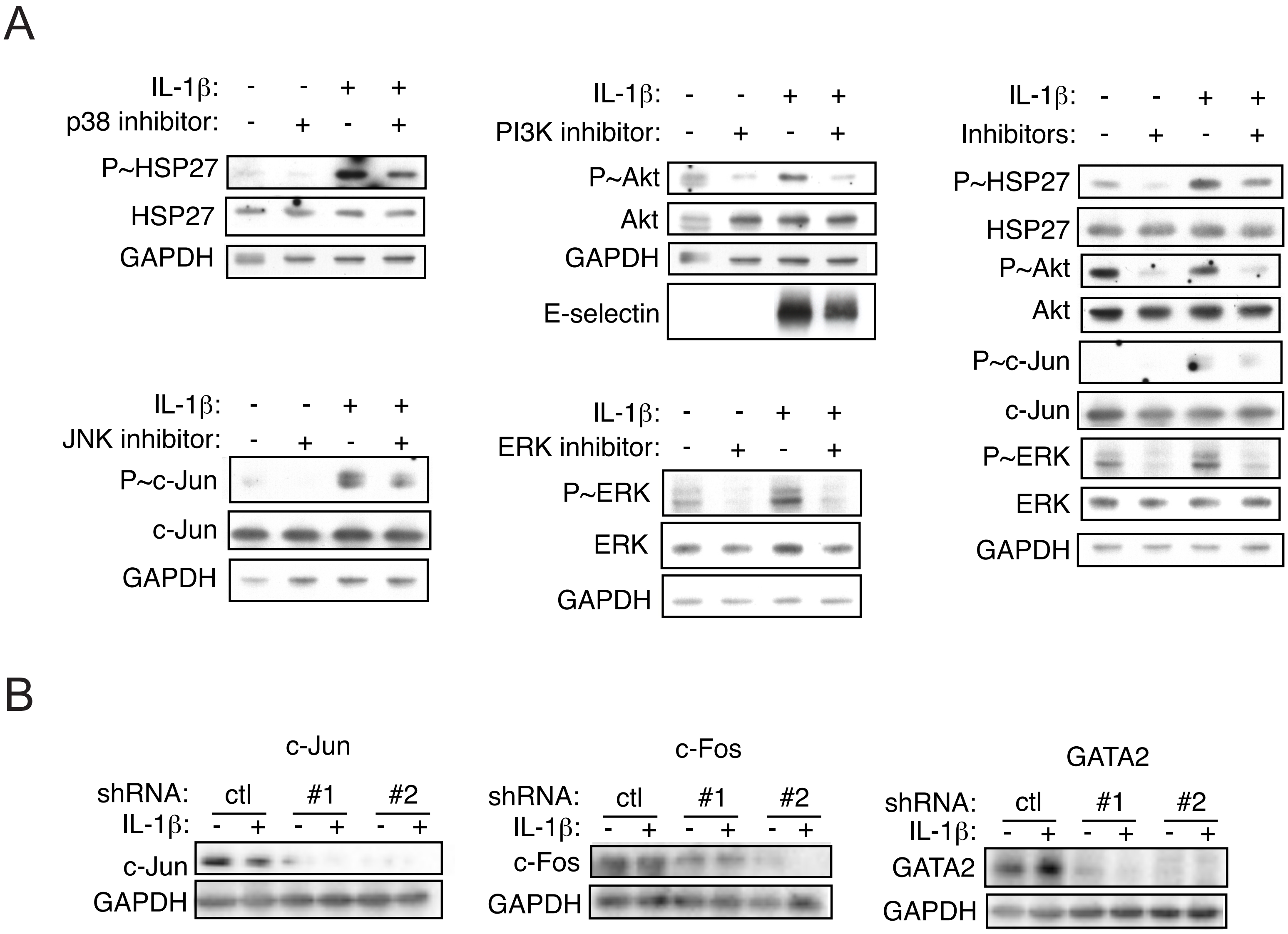

Zhong et al Figure S7

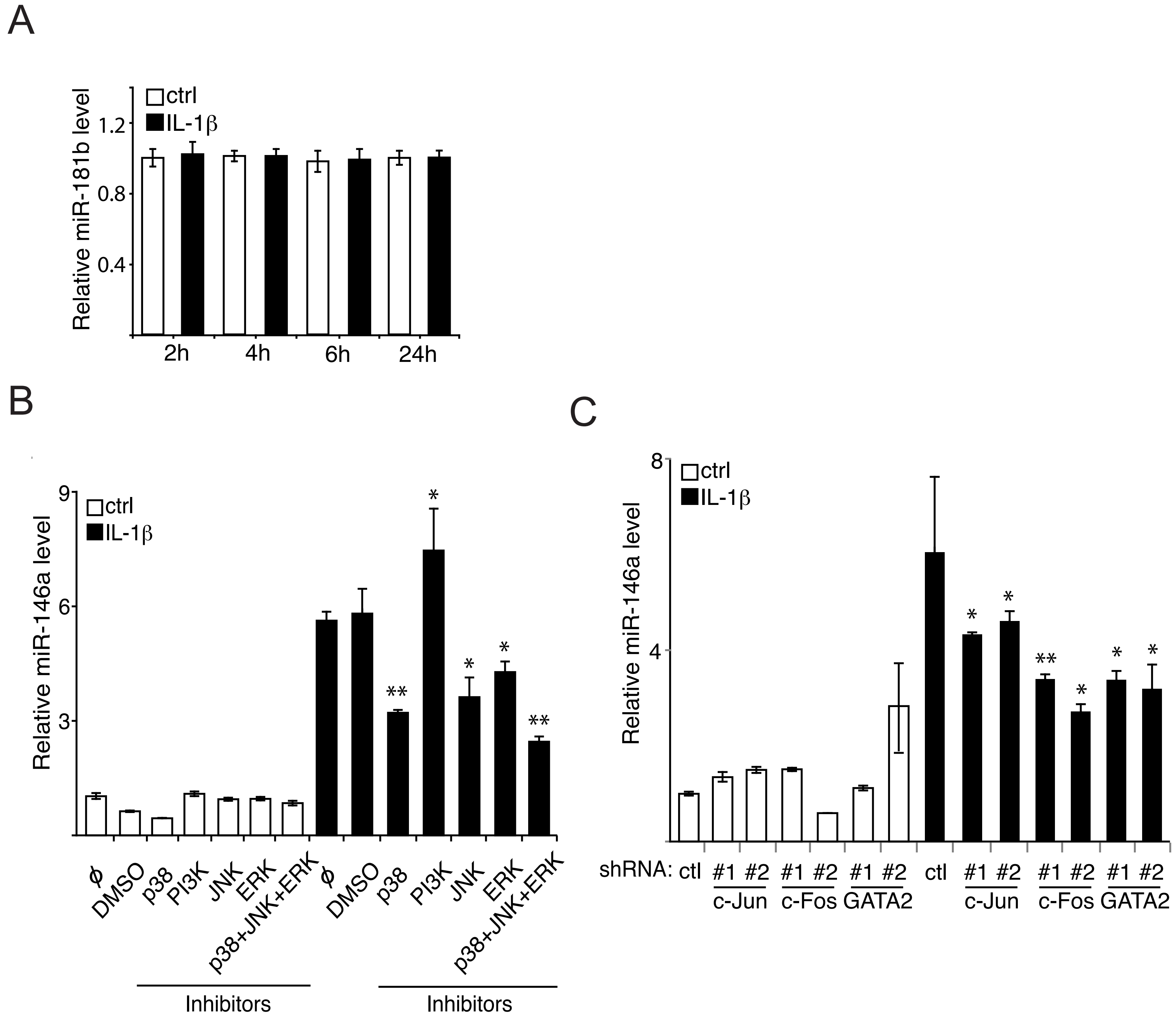

Zhong et al Figure S8

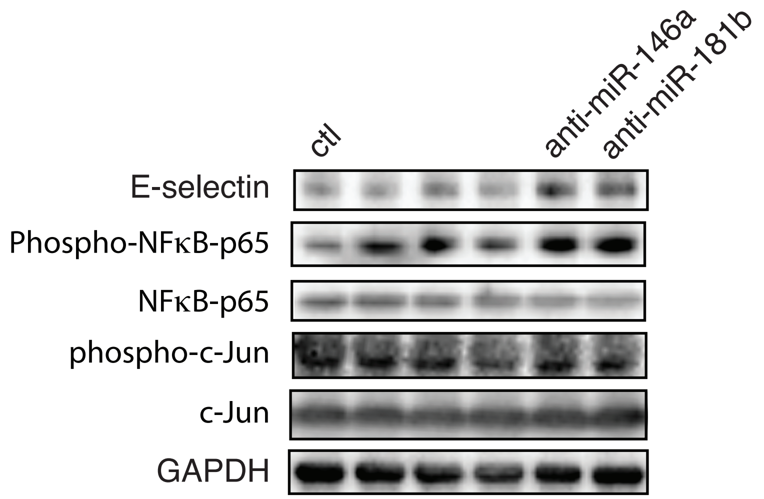

Zhong et al Figure S9
